# Extended charge banking model of dual path shocks for implantable cardioverter defibrillators

**DOI:** 10.1186/1475-925X-7-22

**Published:** 2008-08-01

**Authors:** Derek J Dosdall, James D Sweeney

**Affiliations:** 1 Department of Biomedical Engineering at the University of Alabama at Birmingham, Birmingham, Alabama, USA; 2 Department of Bioengineering at Florida Gulf Coast University, Fort Myers, Florida, USA

## Abstract

**Background:**

Single path defibrillation shock methods have been improved through the use of the Charge Banking Model of defibrillation, which predicts the response of the heart to shocks as a simple resistor-capacitor (RC) circuit. While dual path defibrillation configurations have significantly reduced defibrillation thresholds, improvements to dual path defibrillation techniques have been limited to experimental observations without a practical model to aid in improving dual path defibrillation techniques.

**Methods:**

The Charge Banking Model has been extended into a new Extended Charge Banking Model of defibrillation that represents small sections of the heart as separate RC circuits, uses a weighting factor based on published defibrillation shock field gradient measures, and implements a critical mass criteria to predict the relative efficacy of single and dual path defibrillation shocks.

**Results:**

The new model reproduced the results from several published experimental protocols that demonstrated the relative efficacy of dual path defibrillation shocks. The model predicts that time between phases or pulses of dual path defibrillation shock configurations should be minimized to maximize shock efficacy.

**Discussion:**

Through this approach the Extended Charge Banking Model predictions may be used to improve dual path and multi-pulse defibrillation techniques, which have been shown experimentally to lower defibrillation thresholds substantially. The new model may be a useful tool to help in further improving dual path and multiple pulse defibrillation techniques by predicting optimal pulse durations and shock timing parameters.

## Introduction

### Charge Banking and Charge Burping Models of Defibrillation

More than a century ago, Weiss and Lapique carried early electrophysical experiments to characterize the response of excitable muscle tissue to electrical stimuli[1,2]. In the 1930s, Blair developed a simple resistor-capacitor (RC) lumped sum model to represent the response of excitable tissue to electrical stimuli[3]. This model represents the cell response as an RC circuit that reacts to electrical pulses which is a surrogate for a change in transmembrane potential. More recently, Kroll developed an approach to model the response of cardiac tissue to the capacitive discharge of a defibrillation shock [4]. Kroll's theory of Charge Banking states that charge is built up over the cell membrane and that since the stimulation pulses are capacitive discharges, there is an optimal duration where the membrane voltage of the cardiac cells reaches a maximum. He developed equations describing optimal pulse duration and system capacitance that maximize the response of the model to shocks. Kroll termed biphasic defibrillation modeling with this simple RC model as Charge Burping [5] The second phase of a biphasic shock discharges or 'burps' the stored capacitance and returns the model voltage back to zero as quickly as possible, thereby reducing the recurrence of fibrillation.

The theories of Charge Banking and Burping have led to improvements of several aspects of defibrillation. Internal defibrillation waveform shape [6-9], system capacitance[4], pulse duration [5,10-12], and transthoracic shocks [13-18] have all been improved through the use of this simple model. Animal[10,12,15-17,19-21] and human[8,11,22-24] studies have confirmed the predictive value of the charge banking and charge burping models of defibrillation.

### Multi-path Defibrillation

Cardiac mapping during defibrillation shocks has demonstrated that shocks delivered near the defibrillation threshold (DFT) often fail because excitation wavefronts emerge from areas of low field gradient at sites distant from the shocking electrodes [25-28]. With a shocking coil in the right ventricular (RV) apex, the left ventricular (LV) free wall has relatively low current density and field gradient during a defibrillation shock[29]. In an effort to distribute the current to this area of the heart, a number of recent studies conducted in dogs[30,31], pigs [32-36], and humans [37-41] have used the coronary venous system to spread current to the LV free wall. Several studies have explored the use of an auxiliary shock delivered to an LV electrode [30-33,42,43]. Other studies have included an LV electrode as an additional path for current in parallel with other standard clinical locations[34,37-39,42]. Research has demonstrated that sequential pulses delivered through multiple pathways lowered voltage and energy required for defibrillation[36,44-55].

**Figure 1:**
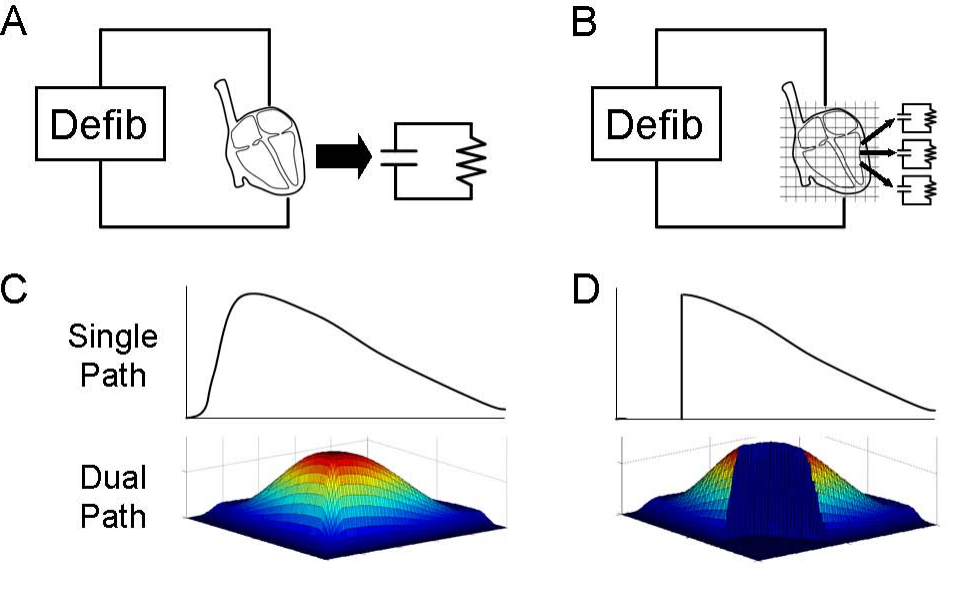
**Overview of the Extended Charge Banking Model for dual-path defibrillation.** (A) The Charge Banking Model estimates the response of the heart to a shock as an RC circuit model. (B) The effect of a shock on many small sections of the heart may be estimated by modeling the response of each section as a separate RC circuit. (C) A weighting function approximating the current distribution through the heart for single path and dual path shocks is combined with the sectioned heart model to predict the response of the whole heart to a shock. See Figures 3 and 4 for additional labels and text for explanation. (D) Successful defibrillation is predicted by determining the effectiveness of single and dual path shocks in causing a minimum threshold model voltage in a critical mass of the heart. See Figure 5 for coordinate labels and text for further explanation.

Many investigators have proposed methods for multi-path and multi-pulse defibrillation, but a method for comparing the relative efficacy of different shocking strategies and pulse timings based on modeling of multipath shocks before resorting to animal or human trials has not been published. We propose an Extended Charge Banking Model for multi-pulse defibrillation which will allow investigators to compare leading edge voltage and energy requirements of dual path shocks as compared to a control waveform. Figure 1 shows an overview of this new model.

## Methods

For a summary of the equations used in the Charge Banking Model and the Extended Charge Banking Model, as well as the equations used to calculate delivered energy and final capacitor voltage (see Appendix).

### Fixing Variables in the Charge Banking and Burping Models

Cardiac tissue time constants for defibrillation, defibrillator capacitor size, and system impedance all affect the behavior of the Charge Banking model. In a study on human patients, Swerdlow and colleagues [22] reported varying values for transvenous defibrillation tissue time constant (also referred to as tissue membrane time constant, τ_m_) between 2.3 ± 0.4 ms and 3.2 ± 0.5 ms, depending on whether leading edge time constant or average current was used to calculate time constant. Irnich states that the chronaxie for defibrillation is approximately 2 ms[56], which can be translated to a τ_m _value of 2.8 ms, by using a relation given by Blair (chronaxie = 0.693 *τ*_m_)[3]. Gold and Shorofsky [57] measured transvenous defibrillation time constant as 5.3 ms. Another study on twenty-three patients reported that chronaxie varied from 1.2 to 12.4 ms, with a mean value of 4.6 ms [11], or a τ_m _of 6.4 ms. Mowrey and colleagues [58] measured transvenous defibrillation τ_m _as ranging from 1.6 to 14.2 ms. Measurements of the tissue time constant vary significantly not only from study to study, but from patient to patient within each study. To keep this work comparable to other studies done with the simplified first order linear model, a value of 2.8 ms is used for τ_m_.

Although system capacitance for commercial ICDs is typically 86–155 μF, the optimal system capacitance has been an issue for debate[59]. Several groups have used Charge Banking and Burping Models of defibrillation to predict more efficient defibrillation with smaller system capacitances[4,60,61]. A larger defibrillator capacitor creates a larger time constant that describes the decay of defibrillation shock (see Appendix). As shown in the equations (see Appendix), a smaller defibrillation capacitor would require a higher initial current to achieve the same response by the model. While optimal system capacitance is still unclear, a fixed system capacitance of 150 μF is assumed for ease of comparison against experimental results and other modeling efforts. Electrode resistance, R_e_, in patients with an RV coil electrode has been found to range from 20 to 80 Ω [40,62-65], with an average value close to 40 Ω[63,64]. During the remainder of modeling results presented in this paper, R_e _is fixed at 40 Ω.

Using the Charge Banking Model for single path defibrillation shocks, optimal first phase duration for a biphasic defibrillation waveform is 4 ms and the second phase optimal duration is 2.2 ms[10]. For development of the Extended Charge Banking Model, these phase durations were used. When comparing the model against experimental data, the pulse durations of the experimental data were used in the model.

### Extended Charge Banking Model

Current density and shock strength are not uniform throughout cardiac tissue during defibrillation [29,66-68]. Regions of high current density exist near defibrillation electrodes while regions of low current density remain at a distance from the electrodes. By segmenting the heart into small compartments, current density may be considered for a small section of tissue. The response of a small section of cardiac tissue can then be examined independently. Current density affecting any small section of the heart may theoretically be anywhere from full shock strength for a section of tissue containing a point source electrode, down to nearly zero amplitude for a location very far from the electrodes. For a control shock, sections of tissue that receive less than full strength shocks would be affected by only a scaled portion of the shock current. Response of the tissue to the shock will then be a scaled version of full strength response.

**Figure 2:**
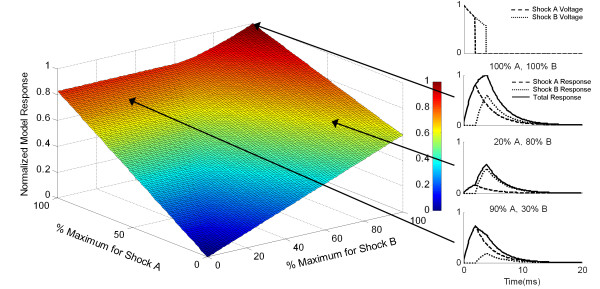
**Responses of tissue receiving varying strength shocks from a sequentially pulsed waveform delivered between two electrode sets (A and B) as the shock strengths for each pulse is varied from 0 to 100% of full strength. **The normalized sequentially pulsed waveform is shown on the top right. The maximum response values for each combination of Shock A and Shock B are shown. Examples of the model response to Shock A, Shock B, and the added responses are shown for 3 combinations of shock strength.

For a dual path shock with two pairs of electrodes, there are potentially sections of tissue that receive 0 to 100% of the shock from one shocking path or set of electrodes, and sections of tissue that receive 0 to 100% of the shock from the other shocking path. The superposition principle allows contributions from multiple shocks to be calculated separately and then added together to determine the overall response (see Appendix). With a sequentially switched waveform delivered from a single capacitor, response for each section of tissue may be calculated using a scaled input current. Response to the Charge Banking Model was calculated for every combination from 0 to 100% of the maximal shock strength for a two path defibrillation shock. The maximum value was extracted from each response, and maximum responses for all values from 0 to 100% for a switched waveform are shown in Figure 2.

### Weighting Function

Since defibrillation current density and potential field gradient are not uniform throughout the heart during defibrillation shocks, a weighting function representing the distribution of current through the mass of the heart was needed. Since it is not possible to measure current density in many locations in the heart directly, potential field gradient is used as an approximation of current density. Several studies have mapped potential gradient fields during single path defibrillation shocks[29,66-68]. These studies examined a number of transvenous shocking paths and shock strength recording techniques. Table 1 shows some of the potential field gradient measurements from these experiments.

**Table 1:** Measured Potential Field Gradient at Defibrillation Threshold

	Lowest Gradient (V/cm)	Highest Gradient (V/cm)	Average Gradient (V/cm)
Tang et al.[66]	2	37	11.7
Witkowski et al.[67]	4.2	30.6	12.9
Tang et al.[29]	3.5–5.8	49.1–104.0	Not given
Chen et al.[68]	7 to 8	99 to 153	Not given

Several characteristics between these mapping studies were considered while determining a weighting function of a potential field gradient distribution for use in the Extended Charge Banking Model approach. First, the measured shock gradient was not zero for any point on the heart during defibrillation shocks given at the 50% successful defibrillation threshold. Each potential field gradient study demonstrated a nonzero minimum gradient recorded throughout the cardiac tissue during shocks. The lowest gradients reported were 5–10% of the maximum measured potential field gradient. Therefore, a weighting function should not contain any points that receive less than 5% of the maximum shock strength. Second, the highest potential field gradients were measured at relatively few points in the heart. The tissue affected by the highest potential field gradient was typically found immediately adjacent to active electrodes. Tissue exposed to high potential field gradients would thus have a relatively small influence in a weighting function. Third, large regions of tissue were exposed to fairly uniform and relatively low shock gradient as compared to the high gradient regions. The average gradient values from these studies tended to be 30–45% of the maximal gradient values. Since none of the measured field gradients were lower than 5–10% of the maximum values, and the average value must fall between 30–45% of the maximum recorded field gradient value, a weighing function must have a relatively high weighing at approximately 20% of the maximum measured field gradient.

**Figure 3:**
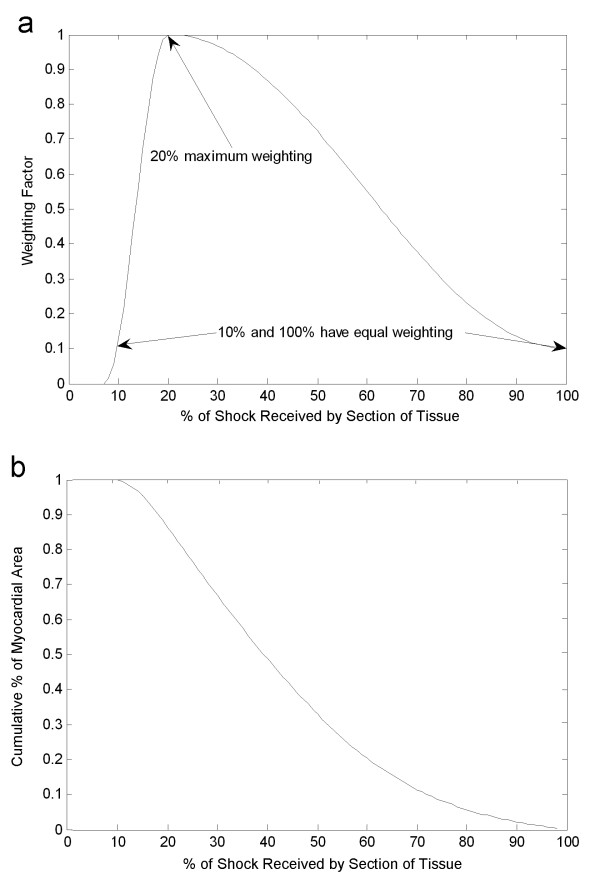
**Weighting function based on published field gradient measurements. **a) Tissue receiving 20% of the shock received the highest weighting (1) while tissue receiving 10% and 100% of the shock received fixed weightings of 0.1. The cumulative weighting distribution (b) demonstrates that while approximately 80% of the tissue receives at least 24% of the full shock strength, only 20% of the tissue receives 60% of the full shock strength.

Based on the observations above, several points were considered fixed while developing the weighting function for use in this model. The highest weighting was fixed at 20% of the maximum shock strength and had a normalized amplitude of 1. Tissue that received 10% and 100% of the maximum shock strength were fixed at a relatively low amplitude of 0.1 and between 8% and 10% the weighting function diminished to zero. Between the fixed points outlined above, the slope of the curve was determined arbitrarily to be a cosine function with an offset rising from 0 to 1 below 20%, and falling from 1 to .1 above 20%. Figure 3a shows the weighting designed with these criteria. Figure 3b shows the cumulative weighting distribution for the amount of tissue that receives any given shock strength. The average shock strength received by the tissue with the weighting distribution presented in Figure 3 was 40% of the maximum shock strength, which is within the range of average values reported in Table 1. This cumulative weighting distribution was remarkably similar to measured distributions of shock strength in animal experiments [29], regardless of the electrode configuration used. Finite element modeling of defibrillation shock distribution with various shocking electrode configurations has also demonstrated similar cumulative shock strength distribution[69]. The weighting distribution shown in Figure 3 was not meant to reproduce exactly the distribution observed during shocks delivered with any single electrode configuration, but was intended to be general enough to represent many internal defibrillation electrode configurations. The proposed weighting function contains many of the basic characteristics observed from diverse mapping studies.

**Figure 4:**
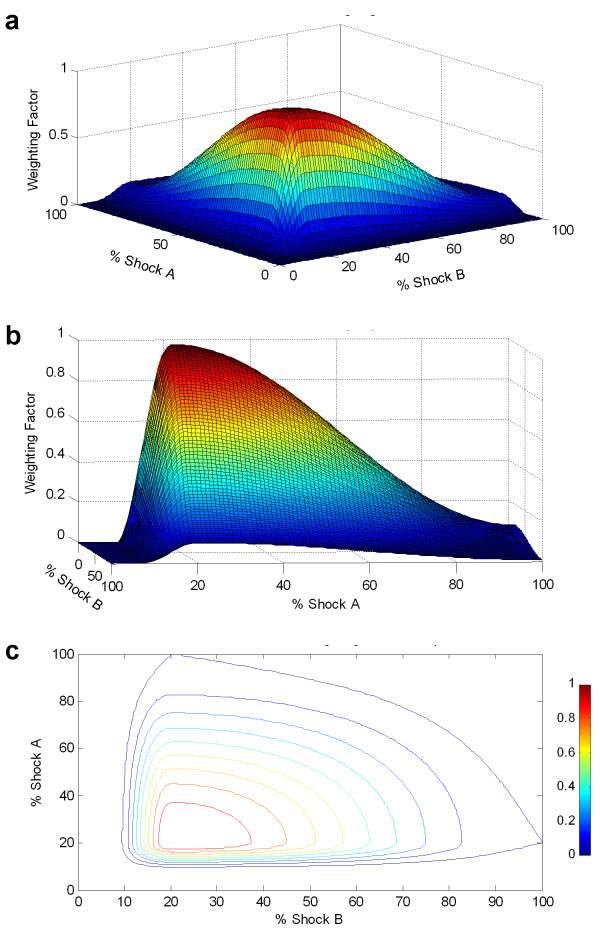
**Alternate three dimensional views of the switched dual path shock waveform weighting function. **The side view (b) shows the same shape as the control waveform weighting function. The bottom figure (c) shows a contour map of the weighting function.

Weighting for a control shock presented in Figure 3 represented the weighting function for single path shocks. Since there are no published results of field gradient measures with sequentially switched dual path shocks, an estimate of the field distribution of a dual path shock was extrapolated from the results of single path defibrillation field gradient measurements. It was assumed that the potential field gradient distribution for both shocking paths was the same, and that the field gradient distributions were independent and randomly distributed through each other. In other words, the first shock field gradient distribution had no effect on the second shock field gradient distribution. To extrapolate this to a weighting for two independent shocking paths, weighting of Shock A was multiplied by the transpose of the weighting of Shock B. Since the shock field distributions for Shock A and Shock B were assumed to be similar, the column vector represented in Figure 3 is multiplied by its transpose, a row vector of the same values. When these column and row vectors were multiplied, a matrix representing the weighting function of two independent paths was calculated. Figure 4 shows weighting for a dual path electrode configuration. As expected, the weighting was zero for all points below 8% shock strength for either A or B. The peak of the distribution was at 20% A, 20% B, and had a weighting of 1. Tissue that received 100% A and 100% B had a relatively low weighting of 0.01.

### Critical Mass

The critical mass theory of defibrillation [70] states that successful defibrillation of the heart is achieved when a shock terminates fibrillation wavefronts in at least 75–90% of the cardiac tissue [69,71]. If only small sections of tissue are not captured, fibrillation wavefronts become too small to be self-sustaining, and ventricular fibrillation is terminated. Using the critical mass criteria and the weighting distribution developed, a DFT may be determined as a function of the maximum shock strength. To measure critical mass with the Extended Charge Banking Model, maximum response of tissue at the critical mass level was calculated. For example, if critical mass was defined as 80%, 80% of the tissue was required to reach a threshold for successful defibrillation. Since absolute transmembrane responses cannot be derived from the Charge Banking model, threshold responses were derived as percentages of maximal responses.

**Figure 5:**
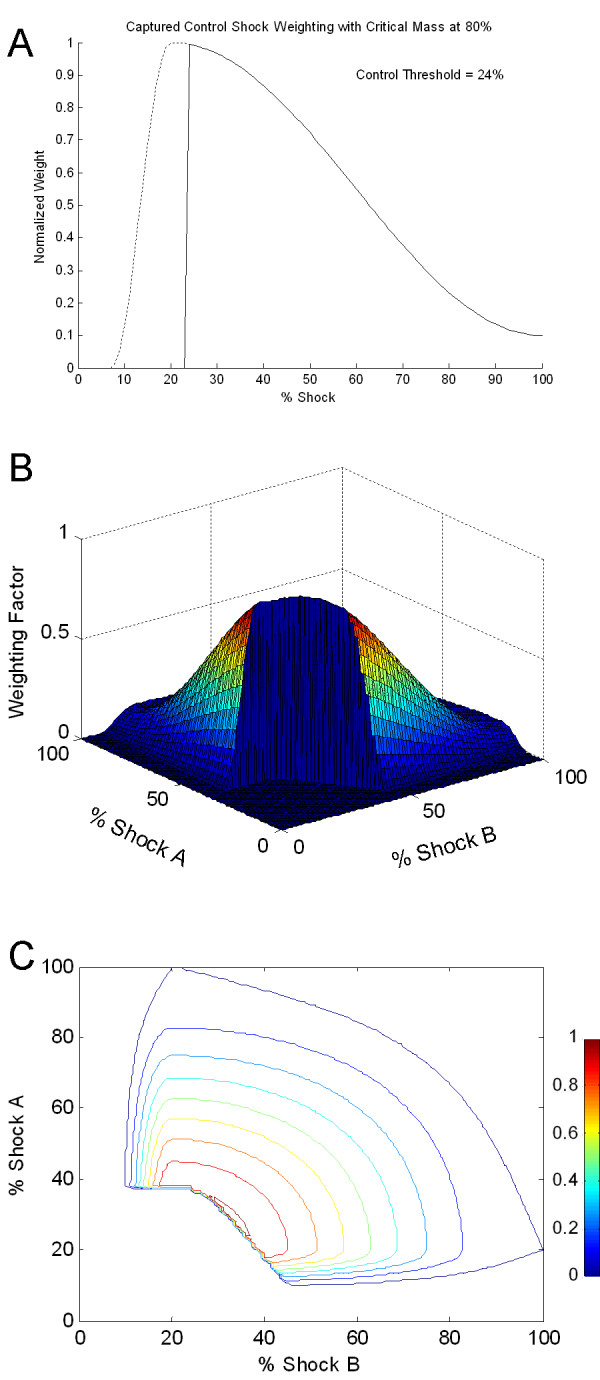
**Critical mass criteria combined with a control shock weighting function.** (A) For a single path shock and a critical mass of 80%, or for 80% of the area under the curve to achieve threshold, tissue that receives 24% of the maximum shock strength must reach threshold for the shock to successfully defibrillate. The area under the dashed line represents the 20% of tissue that would have a subthreshold response to a shock delivered at the DFT. (B) Critical mass criteria combined with a switched shock weighting function. For a critical mass of 80%, the combined response of the tissue must be at least 31% of the combined maximum response for the shock to successfully defibrillate. The removed section represents the 20% of tissue with a response of less than 31% of the maximum response. (C) A contour map of the dual path weighting function (shown in B) with the lowest model voltage 20% response is shown.

For the new model, Control (or single path defibrillation configurations) waveform thresholds were defined as the minimum percentage of the 100% shock strength response observed by a critical mass of the tissue. Initial capacitor voltages that were required for successful capture of a critical mass were calculated. Figure 5A shows the threshold required to capture at least 80% of the weighted tissue with a single path defibrillation shock. To capture 80% of the tissue with a control shock, tissue that received 24% of the maximum shock strength must have a response strong enough to reach threshold. In other words, tissue receiving 24% of the full strength shock would need to be excited sufficiently to terminate fibrillation wavefronts.

Figure 5B and 5C show a switched waveform (discharge from a single capacitor where the shocking path is switched to a second electrode configuration halfway through the first phase) with 20% of the tissue with the lowest response to the defibrillation shock removed. For a switched shock, to capture 80% of the weighted tissue required 31% of the full strength shock to elicit a threshold response. The thresholds for a control waveform and the switched waveform were then compared and normalized to calculate the relative shock strength required for capture with a control shock versus a switched shock. So for 80% critical mass criteria, a switched waveform required only 84% of the voltage that a control shock required.

When DFT reductions were calculated for critical mass criteria ranging from 75%–90%, energy reductions were between 38%–44% regardless of the critical mass criterion used. Table 2 shows the reductions in DFT for each critical mass when comparing a sequentially switched waveform and a control waveform in terms of leading edge current and as delivered energy. Since the critical mass criteria affects the energy savings when comparing control and switched waveforms only slightly when critical mass is 75%–90%, the critical mass was arbitrarily assigned as 80% during the modeling work.

**Table 2:** Shock Reduction for Switched Shock vs. Control with Varying Critical Mass Criteria

Critical Mass	Shock Current Reduction	Shock Energy Reduction
75%	21%	38%
80%	22%	39%
85%	25%	44%
90%	24%	42%

### Comparison of Animal Data and the Extended Charge Banking Model

Results from previously published experimental rapid switching techniques were compared to the Extended Charge Banking Model results. Comparing the model against published results provided an opportunity to validate the predicted model results.

Several studies have been performed that verified the reduction of leading edge voltage and delivered energy by spreading current between pairs of electrodes[36,72]. In each case, both phases of a biphasic envelope (7 ms first phase, 4 ms second phase) were divided into 2 subpulses (2 subpulses of 3.5 ms and 2 ms, respectively), and the subpulses were delivered sequentially to two electrode pairs. In studies by Reighard et al., the leading edge pulse was delivered from an RV coil to an SVC coil, and the second subpulse was delivered from an electrode sutured to the LV epicardial apex to a coil in the outflow tract. Dosdall et al. used two endovascular electrode configurations with the same pulse timing. With the first electrode configuration, the leading edge pulse was delivered from an RV coil to an SVC coil shorted to a subcutaneous hot can electrode, and the second subpulse was delivered from an electrode in the middle cardiac vein to an SVC coil shorted to a subcutaneous hot can electrode. With the second electrode configuration, the first phase subpulse was delivered from the RV coil to the SVC coil, and the second subpulses were delivered from the middle cardiac vein electrode to a subcutaneous hot can electrode. Pulse durations from the experimental work while sequentially switching between a pair of electrode configurations were input into the Extended Charge Banking Model to predict reductions in leading edge voltage and delivered energy.

KenKnight and colleagues conducted a study in dogs delivering an auxiliary shock preceding the primary shock in the coronary venous system[31]. They used 4 ms first phase and 3 ms second phase biphasic shocks as the control shock and as the primary shock for the other configurations tested. In addition to control shocks, DFTs were determined for four other configurations. Monophasic auxiliary shocks (2 ms in duration) from the same capacitor as the primary shocks were delivered to a shocking coil in the left posterior coronary vein 1, 5, 10 and 20 ms before the primary shocks. Shocking patterns used in this study were input into the Extended Charge Banking Model, and reductions in leading edge voltage and delivered energy were calculated.

## Results and discussion

**Figure 6:**
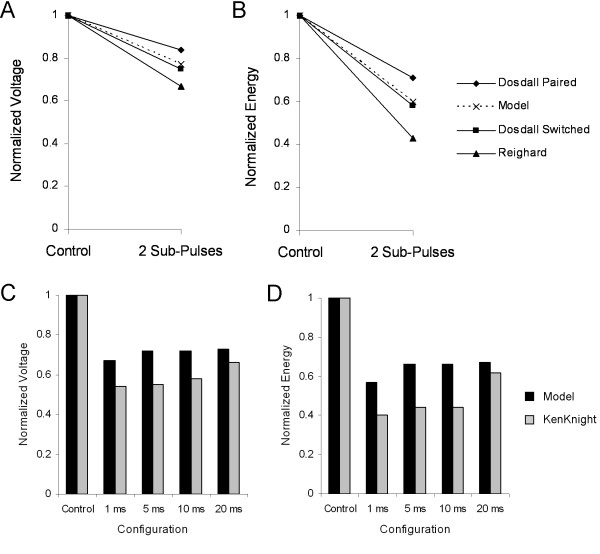
**Comparison of Extended Charge Banking Model to experimental results to experimental findings.** The new model results are compared against Dosdall et al.[36] and Reighard et al.[72] for sequentially pulsed waveforms in terms of leading edge voltage (A) and delivered energy (B). The model results are compared against the findings of KenKnight et al.[31], which consisted of a biphasic waveform preceded by an auxiliary shock from an LV electrode with 1–20 ms between the shocks in terms of leading edge voltage (C) and delivered energy (C). See text for further description of electrode configuration and waveform used.

Figure 6A and 6B shows the normalized leading edge voltage and energy reductions for each of the sequentially switched electrode configurations as compared to a single path control configuration, as well as the voltage and energy reductions as predicted by the Extended Charge Banking Model. All data were normalized to the control for experimental and model voltage and energy. The experimental protocols demonstrated reductions in leading edge voltage of 16%–31%, while the model predicted a reduction in leading edge voltage of 23%. Delivered energy was reduced by 29%–57% in the experimental protocols, while the model predicted a reduction of 40% in energy.

The results from experimental work as described by KenKnight et al. with the auxiliary pulse and the Extended Charge Banking Model are shown in Figure 6C and 6D. The experimental work showed reductions in leading edge voltage of 34%–46% and reductions in delivered energy of 38%–60% depending on the delay between an auxiliary pulse and a primary shock. The Extended Charge Banking Model predicted a decrease in leading edge voltage of 27%–33% and a decrease of 33%–44% in energy.

Figure 2 reveals several important characteristics of switched shock waveform responses. Symmetry about the centerline was not observed due to the sequential nature of the shocks and the discharge of the defibrillation capacitor. Since Shock A led Shock B, the amplitude of the Shock A response without any Shock B response was higher for equal shock contributions. The slope of the Shock A axis response with zero contribution from Shock B was greater than the slope of the Shock B axis response with zero contribution from Shock A. On the left hand side of the figure, there was a region that showed no increase in maximum response while the Shock B contribution rose. Since the model voltage was created during Shock A, response to Shock B had to be greater than the discharge rate of the model from Shock A for Shock B to have any effect on the maximum overall response.

The Extended Charge Banking Model demonstrated sensitivity to pulse duration in the same way that the Charge Banking model demonstrated sensitivity to pulse duration. Figure 2 shows that different pulse durations caused the response to increase until a maximum response was observed. After the maximum response was achieved, longer pulse durations did not increase response, but did continue to expend energy while the pulse was active.

Sequential shocks from two pathways experimentally demonstrated a range of voltage and energy reductions (as shown in Figure 6A and 6B), even when identical pulse timings were used. The variations in voltage and energy reductions were likely due to the different current distributions established with the different electrode configurations used. Since the Extended Charge Banking Model did not take into account variations in electrode configuration, the reductions in voltage and energy predicted by the model did not precisely match any of the experimentally observed reductions. However, the model did predict reductions in voltage and energy that are well within the observed experimental ranges.

When compared against the experimental results of KenKnight et al., the Extended Charge Banking Model did not predict the same reductions in voltage and energy quantitatively as the experimental data with an auxiliary pulse, but it did correctly predict that the greatest reductions in voltage and energy would be observed with the smallest delay between the auxiliary and primary pulse. Auxiliary pulse separation had an effect on the Extended Charge Banking Model because the time constant used in the model (τ_m _= 2.8 ms) was on the same order as the delays between shocks. The smallest separation used by KenKnight et al. was 1 ms, which allowed approximately 30% of the induced model voltage from the auxiliary shock to discharge before the primary shock was delivered. At 5 ms, which was almost two time constants, approximately 80% of the first shock response had dissipated. Therefore, the model predicted that the most effective switched waveform were waveforms with no delay between pulses.

Both the model and the KenKnight et al. study demonstrated that an auxiliary shock delivered 20 ms before an initial shock could lower DFTs substantially (Figure 6C and 6D). After near DFT level shocks, there is a period of 30–60 ms during which no electrical activity is observed[27,73-76]. During this quiet period, a second shock from a different electrode set may have the ability to independently capture and effectively defibrillate tissue that was distant from the first electrode set. So while neither the auxiliary shock nor the primary shock by itself was capable of defibrillating the entire heart, the combined effects of both shocks were able to excite enough tissue between both electrode sets to defibrillate the heart. Closely timed sequential shocks (shocks delivered with much less than τ_m _between shocks) had a cumulative effect on model response because the first shock response had not discharged before the second shock began. Pulses delivered in rapid succession (<1 ms between shocks) used the spatial and temporal advantages provided by two shocks, while shocks delivered with a longer pause (5–20 ms delay) only took advantage of the spatial effects of two shock.

The Extended Charge Banking Model may be used to compare large numbers of different defibrillation shock waveform strategies and parameters. Since animals do not tolerate large numbers of VF episodes and shocks well, it is not possible to efficiently study more than approximately 6–8 waveforms within a single study. The model could be useful for predicting a few optimized waveforms, and experiments could be carried out in a controlled way to verify that predicted DFT reductions are observed in animal studies. The model also could be made more specific to a particular shock configuration by changing parameters such as system capacitance, electrode resistance, pulse durations, and weighting functions. Tailoring the model to these specific parameters would likely lead to more accurate results and predictions as compared to experimental work.

Efficacy of defibrillation shocks was predicted in this model by the amplitude of the response of the modeled tissue to the first phase of the shock. Biphasic shocks have been shown to be more effective than similar strength monophasic shocks[68], and the optimal second phase duration would remove the model voltage built up on the first phase[9,10]. The model may be used to predict the optimal second phase shocks to remove the voltage built up on each of the first phase shocks delivered with each shocking path.

### Limitations of the Extended Charge Banking Model

While the Extended Charge Banking Model provides insight into effective multipulse defibrillation strategies, the model does not quantitatively reproduce experimental results in all cases. It does not take into account heart size, electrode configuration, disease states, pharmaceutical side effects, or other factors that can change defibrillation thresholds. Average values for potential field gradients, tissue time constant, system impedance, and system capacitance were used. Each of these parameters could potentially alter model performance. In an effort to better reproduce experimental results or to predict results based on known parameters, the model could be tailored to use configuration-specific information such as the actual defibrillator capacitance, programmed pulse durations, or weighting functions derived from mapping studies of sequentially pulsed dual path electrode configurations.

The weighting function was developed from reasonable assumptions based on mapping studies of potential field gradient during defibrillation shocks. Mapping studies were conducted with various electrode configurations, but to understand potential field gradients for different lead systems, mapping of specific electrode configurations of interest could be conducted. Electrode configuration may significantly alter the potential field gradient, and the resulting potential field gradient measures may provide different guidelines for the development of an appropriate weighting function. Weightings derived specifically from electrode configurations being modeled may provide more accurate results. While field gradient is an important factor in defibrillation shock success, other factors have also been shown to play important roles. Fiber curvature and virtual electrode effects were not accounted for in the Extended Charge Banking Model. A more accurate weighting algorithm might be based on mapped tissue response, rather than field gradient during defibrillation shocks. Near DFT failures tend to initiate in areas of low field gradient, but the probabilistic nature of defibrillation indicates that success is determined by more than field gradient of defibrillation shocks.

The charge banking model uses the response of an RC circuit as a substitute for transmembrane voltage. However, this model is representative only of the behavior of cardiac tissue in response to shocks. The charge banking model and, by extension, the Expanded Charge Banking Model do not attempt to represent specific mechanisms such as virtual electrode effects, electroporation, critical points, or others that have been shown to play a role in defibrillation.

## Conclusion

The Expanded Charge Banking Model of defibrillation predicted reductions in leading edge voltage and delivered energy for sequential pulsed shocks with two electrode sets that were similar to reductions observed experimentally. It also correctly predicted that leading edge voltage and delivered energy were minimized when an auxiliary shock and a primary shock were delivered as closely together as possible. This simple model may be used to improve defibrillation waveforms and shock timing for multipulse defibrillation techniques with dual shocking paths.

## Competing interests

The authors declare that they have no competing interests.

## Authors' contributions

DD conceived the model design, was responsible for the implementation of the model, and was primarily responsible for the preparation of all aspects of the manuscript. JS advised and consulted with DD in all aspects of the design, reviewed results of the model, aided in the interpretation of the results, and has approved the final version of the manuscript. All authors read and approved the final manuscript.

## Appendix

### Charge Banking and Burping Model Equations

Walcott and colleagues[10] used Kroll's basic framework to optimize pulse duration of defibrillation shocks. For a monophasic truncated exponential waveform, they described the cardiac response to a capacitive discharge defibrillation shock with two equations. One describes the charging of the capacitor in the RC circuit while the pulse is applied:

(1) $$\begin{array}{cc} V(t)=\frac{I_{o}\gamma }{C_{m}}e^{-t/\tau_{m}}\left(e^{t/\gamma }-1\right) & 0\leq t\leq d \end{array}$$

And a second equation describes the decay of the response after the truncation of the stimulation:

(2) $$\begin{array}{cc} V(t)=\frac{I_{o}\gamma }{C_{m}}e^{-t/\tau_{m}}\left(e^{d/\gamma }-1\right) & t>d \end{array}$$

(3) $$\text{Where }\gamma =\frac{\tau_{s}\tau_{m}}{\tau_{s}-\tau_{m}}$$

*τ*_*m *_is the tissue membrane time constant (as described previously in the text), and *τ*_*s*_, the system time constant, is defined as:

(4) *τ*_*s *_= *R*_*e*_*C*_*s*_

where *R*_*e *_is the electrode impedance and *C*_*s *_is the defibrillation system capacitance. *I*_*o *_was the initial current delivered from the defibrillation system at the leading edge of the shock. Shock duration was shown as d, and *C*_*m *_was a scaling factor, which was set to 1.

To calculate the effects of switching between electrodes during a shock, the responses of the model to each portion of the shock were summed to determine the net response. To calculate the model response of a section of tissue to a pulse delivered between two electrodes, the first subpulse, or Shock A, response was calculated using Equation 1 above. When the second pulse, or Shock B, began, the second phase charging response was added to the first phase discharging response to determine the overall model response. The combined first phase response can be determined by:

(5) *V*(*t*) = *V*_1_(*t*) + *V*_2_(*t*)   0 ≤ *t*

Where:

(6) $$\begin{array}{cc} V_{1}(t)=\frac{I_{o}\gamma }{C_{m}}e^{-t/\tau_{m}}a\left(e^{t/\gamma }-1\right) & 0\leq t\leq d_{1} \end{array}$$

(7) $$\begin{array}{cc} V_{1}(t)=\frac{I_{o}\gamma }{C_{m}}e^{-t/\tau_{m}}a\left(e^{d_{1}/\gamma }-1\right) & t>d_{1} \end{array}$$

and

(8) *V*_2_(*t*) = 0   0 ≤ *t*<*d*_2_

(9) $$\begin{array}{cc} V_{2}(t)=\frac{I_{s}\gamma }{C_{m}}e^{-t/\tau_{m}}b\left(e^{t/\gamma }-1\right) & d_{2}\leq t\leq d \end{array}$$

(10) $$\begin{array}{cc} V_{2}(t)=\frac{I_{s}\gamma }{C_{m}}e^{-t/\tau_{m}}b\left(e^{d/\gamma }-1\right) & t>d \end{array}$$

Where *a *is the Shock A scalar from 0 to 1 that represents the amount of current from Shock A that the particular section of tissue receives. The Shock B scalar, *b*, is also a scalar between 0 and 1 that represents the amount of current from Shock B that the tissue receives. The time at which Shock A terminated is *d*_1_, *d*_2 _is the time at which Shock B began, and *d *is the time at which Shock B terminated. *I*_*s *_is the initial current delivered by the defibrillation capacitor at the beginning of Shock B. The net effect of the shocks is calculated as the sum of the passive responses. The arbitrary constant, *C*_*m*_, was set such that the results were normalized to the maximum value of *V*(*t*).

If Shock A and Shock B are sequential (i.e., there is no overlap or time gap between the shocks) and delivered from the same capacitor, the equations simplify to:

(11) $$\begin{array}{cc} V(t)=\frac{I_{o}\gamma }{C_{m}}e^{-t/\tau_{m}}\left[a\left(e^{d_{1}/\gamma }-1\right)+b\left(e^{t-d_{1}/\gamma }-1\right)\right] & d_{1}<t\leq d \end{array}$$

(12) $$\begin{array}{cc} V(t)=\frac{I_{o}\gamma }{C_{m}}e^{-t/\tau_{m}}\left[a\left(e^{d_{1}/\gamma }-1\right)+b\left(e^{d-d_{1}/\gamma }-1\right)\right] & t>d \end{array}$$

Where and *d*_1 _is the time at which the Shock A terminated and Shock B commenced and *d *is the time at which Shock B terminated.

### Energy Calculation

Energy discharged during a defibrillation shock was calculated using the following equation:

(13) $$E=\frac{1}{2}C_{s}(V_{I}^{2}-V_{F}^{2})$$

where E is energy delivered, C_s _is defibrillation system capacitance, V_I _is initial capacitor voltage, and V_F _is final capacitor voltage. Final capacitor voltage can be found using:

(14) $$V_{F}=V_{I}e^{d/\tau_{s}}$$

where d is the duration of the entire shock and τ_s _is the system time constant as described by Equation 4.
